# Eliciting culturally and medically informative family health histories from Marshallese patients living in the United States

**DOI:** 10.1002/jgc4.1249

**Published:** 2020-04-28

**Authors:** Karli Blocker, Henry Gene Hallford, Pearl McElfish, Noelle R. Danylchuk, Lori Williamson Dean

**Affiliations:** ^1^ Department of Genetic Counseling College of Health Professions University of Arkansas for Medical Sciences Little Rock Arkansas; ^2^ Clinical Genetics Stanford Children’s Health Specialty Services San Francisco California; ^3^ Department of Pediatrics Section of Genetics College of Medicine University of Oklahoma Health Sciences Center Oklahoma City Oklahoma; ^4^ Department of Internal Medicine College of Medicine University of Arkansas for Medical Sciences Fayetteville Arkansas

**Keywords:** cultural barriers, disparities, family history, language barriers, Pacific Islander Health, underrepresented populations

## Abstract

The United States (U.S.) resident Marshallese population is growing rapidly. Subsequent to this growth, Marshallese patients experience language and cultural barriers when attempting to access medical care in the U.S. This study: (a) documents how the Marshallese refer to biological and adopted family members; (b) identifies barriers encountered by Marshallese when seeking medical care; and (c) describes effective communication strategies for healthcare providers to use when treating Marshallese patients. Six key informant interviews were conducted in English with bicultural (U.S. and native Marshallese) informants, the majority of whom were women who worked in a healthcare setting. Participants were recruited through the Center for Pacific Islander Health in Arkansas and through personal contacts within the Marshallese community. Based on the study findings, examples of how providers can make genetic services more accessible and meaningful for Marshallese patients are also provided. This study is particularly relevant to genetic counselors as the number of Marshallese patients and families needing their services is growing.

## 1 INTRODUCTION

Obtaining an accurate, multigenerational, family health history (FHH) is an essential component of the genetic evaluation. The process of engaging the patient/patient's family and documenting the FHH facilitates rapport (Bennett, 1999), which is critical as the patient–provider relationship is central to the profession's model of care (Veach, Bartles, & LeRoy, 2007). The FHH also allows genetics providers to identify patients who may be at risk for developing heritable genetic disorders (Doerr & Teng, 2012; Yoon, Scheuner, Jorgensen, & Khoury, 2009). And finally, the FHH can help ensure that patients and their family members are informed about and/or receive the most appropriate care for their condition.

Minority and immigrant populations may be less likely than others to receive a comprehensive FHH as health disparities are well documented in these populations (Armstrong, Ravenell, McMurphy, & Putt, 2007; Gamble, 1997; Williams & Hampton, 2005). Health disparities among these populations are perpetuated by a lack of insurance and the inability to pay for healthcare, language barriers, distrust, cultural differences, and providers’ lack of cultural competency training. The FHH could help to overcome some of these barriers by providing a snapshot of the family's overall health burden and informing the presenting patient not only about his or her own health issues, but also focusing attention on areas of health concerns across the family, which may influence other family members’ decisions to seek healthcare services.

Marshallese patients’ access to and trust in the U.S. healthcare system is particularly compromised, due to their complicated history with the United States government. The U.S. government was granted temporary control of the Marshall Islands in 1944 and, subsequent to this, the Republic of the Marshall Islands (R.M.I.) became the primary site for nuclear weapons testing (Barker, 2004). From 1946 to 1958, the U.S. military detonated 67 nuclear bombs in the R.M.I., resulting in high levels of radiation on many islands and the complete destruction of six islands (Barker, 2004). The largest bomb, called Bravo, created a mushroom cloud that expanded to 25 miles in diameter and was 1,000 times more powerful than either bomb dropped on Hiroshima or Nagasaki (Barker, 2004). Moreover, the U.S. nuclear weapons testing in the R.M.I. created an opportunity for the U.S. military establishment to explore the impact of radiation on humans and on the surrounding environment (Barker, 2004).

Many Marshallese believe the U.S. government chose not to relocate the native inhabitants of the islands contaminated by nuclear testing so as to increase the number of people exposed, allowing the U.S. government to more closely examine the impact of radiation exposure (Barker, 2004). *Project 4.1* was implemented by the U.S. military in 1982, about 30–40 years after the bombings, to conduct research about the effects of radiation exposure on the Marshallese people, largely without their knowledge or consent (Barker, 2004). The nuclear weapons testing in the R.M.I. and subsequent research conducted on the Marshallese population has resulted in distrust in the U.S. government and its healthcare system, which continues to this day.

As a result of more than 12 years of above‐ground nuclear testing, residents of the Marshall Islands experience a wide range of negative health effects due to direct and indirect effects of radiation exposure (Simon, Bouville, Land, & Beck, 2010). Due to contamination of the islands’ food and water supplies, the population's diet also drastically changed (Barker, 2004). The U.S. government imported canned and processed foods for the Marshallese as a safer alternative to the local, contaminated foods (Barker, 2004). While their traditional diet consisted primarily of fresh fish and vegetables, the foods provided by the U.S. were high in sodium and fat, resulting in poorer health for the indigenous Marshallese population (Barker, 2004).

As a result of the Compact of Free Association (COFA) between the U.S. and the R.M.I. in 1986, nearly half the population of the Marshall Islands has moved to, and are living, in the U.S. The largest Marshallese U.S. population concentrations are in Hawaii and Northwest Arkansas, which have nearly tripled from 2000 to 2010. These population numbers are based on U.S. Census data, which likely underestimate the actual numbers of Marshallese immigrants into these areas (McElfish, Hallgren, & Yamada, 2015).

Typically, when European Americans are asked to provide health information about their family members, their answers reflect a nuclear organizational schema, consisting of two discrete sets of grandparents, and a mother and father—though increasingly these may live independently—and their children. The typical Marshallese family structure, however, can be quite different. Rather than a nuclear family arrangement, Marshallese families often include extended family members; parents, grandparents, aunts, uncles, and cousins all of whom may live in the same household (Barker, 2004). This multigenerational, nonnuclear family structure is common to many traditional, non‐Euro‐American cultures. For example, a study of Native American families, which in many ways are similar to Marshallese families, found that Native American children may be cared for by extended family, or even nonbiologically related community members (Weaver & White, 1997). The differing family dynamics experienced by members of many non‐Euro‐American families often cause confusion and misunderstanding when healthcare workers conduct a FHH and attempt to establish the biological relationships between family members.

Despite the abundance of primary and specialty medical care services in Northwest Arkansas, a limited number of trained Marshallese clinical interpreters are available. This results in interpretative services not always being present in clinic. Unfortunately, even when these services are available, significant challenges remain. Primary among these is the fact that many common medical and anatomical terms have no cognate in the Marshallese language. This presents a significant challenge, even to well‐trained interpreters, when explaining complex medical issues to patients, who are often not familiar with basic medical practices (Williams & Hampton, 2005). Also, American healthcare providers often are not sufficiently trained in cultural competency, especially with less common ethnic populations and, therefore, are unable to recognize minority patients’ lack of understanding during medical interviews (Williams & Hampton, 2005). The combined effect of frequently inadequate translation services, insufficient cultural competence, and the typically fluid Marshallese family structure becomes clear when healthcare providers attempt to obtain a FHH. This is especially troubling for genetic service providers, as the inheritance patterns of disease, which can be identified through an accurate FHH, are fundamental to the identification and management of heritable and genetic conditions.

### 1.1 Purpose of the study

This study attempted to clarify the frequent misunderstandings that occur as a result of linguistic, cultural, and family structure differences between U.S. genetics healthcare providers and their Marshallese patients by: (a) documenting how the Marshallese refer to family members; (b) identifying cultural and linguistic barriers likely to impact obtaining an accurate genetic family history from Marshallese patients; and (c) identifying effective communication strategies for clarifying kin relationships and obtaining an accurate genetic family history from Marshallese patients. With the growing number of Marshallese residents in the U.S. (Duke, 2014), there is a clear need for developing new strategies for improving the patient–provider relationship with this population. In clinical genetics, the relationship is strengthened by taking a culturally appropriate FHH, which requires a better understanding of Marshallese culture and the family dynamics common to that culture. This study provides insight into how the Marshallese family experience can be improved during genetics clinic visits.

## 2 METHODS

### 2.1 Overview

After seeking and obtaining Institutional Review Board (IRB) approval from the University of Arkansas for Medical Sciences (IRB #206536), this descriptive/interpretative study employed a semi‐structured, in‐depth interview methodology (following Beck, 1994; Broom, 2005; Charmaz, 2006) to identify the knowledge, feelings, beliefs, attitudes, and experiences of Marshallese community members about the difficulties they have experienced when using healthcare services available to them and about their understanding of the relationship between the terms they use to identify family members and the biological relationships between those family members. All willing participants provided verbal informed consent, in English, before being interviewed. The consent process was audio recorded. Participation was voluntary and strictly confidential. The interviews were conducted between November 2017 and January 2018 and took place either at the participants’ places of work in Northwest Arkansas, or, in one case, was conducted over the telephone. Participants were interviewed individually. Due to the open‐ended nature of the questions, interviews ranged from 25 to 60 min in length.

### 2.2 Participants

Prospective interviewees were identified and approached by members of the research team with the assistance of the Center for Pacific Islander Health, Marshallese Education Initiative, Arkansas Coalition of Marshallese, and through personal contacts within the Marshallese community. Moreover, prior to project initiation, the study was presented to, and approved by the Center for Pacific Islander Health's advisory committee and all subject recruitment was done with their consent. Twenty persons were invited to participate in face‐to‐face interviews. Of these, 12 did not respond to the invitation, and two others were unable to schedule an interview time.

Participants were six Marshallese adults who, were native Marshallese speakers also fluent in English, were current residents of Northwestern Arkansas and had lived in the U.S. for at least five years, and actively participated in both American and Marshallese cultures. Five were born in the R.M.I. and one, though born in the U.S., had lived for an extended period of time in the R.M.I. Five were female and one was male. All interviewees were either directly involved in providing healthcare to the Marshallese community, or were nonmedical community leaders. Given the limited number of community leaders and healthcare providers in this community, participants’ specific roles and duties and their demographic characteristics cannot be provided as this would likely identify them.

### 2.3 Procedures

Interviews were conducted in English, by a non‐Marshallese graduate student in the Genetic Counseling Program at the University of Arkansas for Medical Sciences (UAMS), under the direction of L.W.D. As neither were Marshallese or fluent in the language, a prominent Marshallese community member, trained Marshallese clinical interpreter for the UAMS’ Center for Pacific Islander Health and Community Liaison for the Heartland Regional Genetic Services Network, assisted in arranging and facilitating the interviews and was available during all of the interviews to help with any logistical or linguistic issues that might arise. All interviews were conducted in English, in a private location and participants were individually interviewed.

Interview questions were designed to encourage interviewees to provide comprehensive narrative responses concerning the nature of the Marshallese family structure and how Marshallese persons refer to family members, as well the identification of specific cultural and linguistic barriers that are likely to impact the collection of accurate genetic family history information and effective communication strategies for clarifying kin relationships. The interview guide was developed by K.B., L.W.D., H.G.H., and N.R.D., then reviewed by Marshallese research staff at the Center for Pacific Islander Health. The question guide used in this interview is available in Appendix A as a supplementary file.

These interviews were audio recorded, de‐identified, and then transcribed. Transcription was conducted either by the study staff or outsourced to a secure commercial service, which practices strict privacy policies and requires transcribers to sign nondisclosure agreements.

### 2.4 Data analysis

De‐identified transcripts of participant interviews were analyzed following specific procedures commonly used in qualitative research (Beck, 1994; Birks & Mills, 2011; Charmaz, 2006; Patton, 2002; Smith, 1998). After multiple readings, the PI and two coauthors independently analyzed and coded participant responses within the transcripts, which represent interviewees’ thoughts, feelings, and beliefs, and were responsive to the study's specific aims (Saldana, 2009).

After multiple readings, the analysts identified recurrent themes (i.e., themes which occurred either across many interviews or was a dominant theme within one interview) and direct quotes were identified which expressed these themes (Braun & Clarke, 2006). Analysts then compared, discussed, and agreed upon the common themes. Once each transcript review was completed, analysts met to discuss the codes, and then, returned to the data for further analysis. Inter‐rater reliability was not formally assessed. The results of this iterative process were then presented to and discussed by the student's research committee, which included researchers in and members of the Marshallese community.

## 3 RESULTS

### 3.1 Identified themes

Careful analysis of respondent transcripts identified multiple themes addressing each of the study aims. The three themes responsive to the first aim, which was to characterize Marshallese family structure as it pertains to the FHH, were: (a) the overall construct of Marshallese families; (b) the way in which Marshallese people identify with their home island and with their family through maternal lineage; and (c) the terminology used to identify family members (Table 1).

**Table 1:** Identified themes

Aims	Aim 1: Document how the Marshallese refer to family members	Aim 2: Identify barriers that may impact obtaining an accurate FHH	Aim 3: Identify effective communication strategies to obtain accurate genetic family history from Marshallese patients
Theme 1	Overall construct of Marshallese families	Perceived language barriers	Simple greetings/ introductions help build strong bonds/trust
Theme 2	Personal identification with home island and maternal lineage	Perceived cultural barriers	Provider should explain the rationale of actions during a visit
Theme 3	Terminology used to identify family members		

The themes addressing the second aim, which was to identify barriers likely to impact obtaining an accurate genetic family history from Marshallese patients were perceived language and cultural barriers. Examples of these barriers include lack of trained interpreters and the community's requisite word‐of‐mouth assessment to determine whether or not potential patients will seek American healthcare or a particular provider.

For the final aim, which was to identify effective communication strategies for clarifying kin relationships and obtaining an accurate genetic family history from Marshallese patients, two major themes emerged: key, simple efforts providers can make when first meeting the patient and the provider explaining rationale of each step/action during the visit.

During the conduct of the interviews and the analysis of the interview transcripts, several key Marshallese words were identified, which helped to define and clarify interviewees’ understanding of the concepts under study. These words and their English translations are provided in Appendix B as a supplementary file.

### 3.2 Themes addressing aim 1

All participants stressed the importance of family and noted that families are large and include extended family members. Participant 6 noted, “We do believe in really extended families. We go back four or five generations and we'll still look after each other.” All participants mentioned that family includes the people who grew up around you and were from the same island. Participant 2 explained, “To me and my culture, it means the whole extended family. It also means the people that I grew up around with [*sic*], around the village or the “*weto*” [pronounced wah‐too], or the places that I lived around.”

Another commonality found in all participants was that family also included people who were not related by blood. These individuals could be friends, members of their community, members of their church, colleagues from work, and simply people who care for one another. One participant specifically stated that family “doesn't have to be blood relations” (Participant 1). A few participants also explained that their community, and therefore, their family, does not just include people who are Marshallese. Participant 4 stated that family is “not only just my race, I consider them families [*sic*], but it could be other race[s].”

Another relevant topic identified within this theme was that of the Marshallese perception of adoption. Every participant stated that adopted family members are not viewed any differently than family members who come from their biological lineage. In fact, Participant 5 explained, “When you adopted the child, you…never say ‘oh, she's adopted.’ Like how Americans really differentiate that [*sic*].” It is important to note that adoption is common in the Marshallese culture and will typically occur within families. Several participants mentioned that siblings will adopt each other's children and raise them openly. Participant 6 stated, “You're already in the bloodline, even though you're adopted. You already have your share as far as for the lands or the heritage…there's not meaning for [adoption] in the Marshall Islands.” This participant also gave an explanation of why the Marshallese will allow other family members to raise their children. Some individuals struggle with infertility and are unable to have children of their own. This is important in the Marshallese culture since eventually children will care for their parents as they age and need assistance. When a family member gives another their child, Participant 6 explained, “They usually say ‘*ajej in mona’* [pronounced AH‐jeej EN MOH‐nah]. It literally means passing a support. And they'll give [the child] to you and say okay, here is your ‘*jokon’,* [pronounced joh‐KAHN] [which] literally means cane.” The Marshallese allow another person to raise their biological child because they know that others will need the help later on. Although all participants said adopted family members were not viewed any differently than biological family members, Participant 1 mentioned that the Marshallese word for adopted is “*kajiriri”* [pronounced kah‐jeh‐REE‐ree].

Several participants also mentioned how their individual and family identity is defined by their maternal family lineage and island of origin. Participant 5 explained “Family is everything…There's a saying in my language, ‘if you lose your mom, you lose everything, you lose connection.’” She continued, “…as a daughter it was my job…to take care of my mom. So [now] that my mom is gone…I feel like it is my job to…make sure that [my younger siblings] are taken care of, even though they are grown up.” It is clear that this sense of family and caring for one another continues throughout life, since the Marshallese family bond is so important to them. In the Marshallese culture, the maternal lineage and land are important, as the land is inherited for generations through the maternal side, which creates a great sense of identity. Participant 2 explained, “We're connected to the land. That's how we live, that's how we act and define ourselves. We have what we call ‘*weto*’ [pronounced wah‐too] which is the piece of land that your family has inherited through generations, and that island or atoll…that's where you identify yourself as being from.”

The family dynamic of the Marshallese is further explained by the terms used to identify family members. While in the English language, mother, father, aunt, uncle, brother, sister, and cousin all denote specific relationships, the Marshallese may use some of these words interchangeably. Therefore, asking, “Who is your mother?” can be more complicated for Marshallese patients. Participant 4 explained, “I call my other aunts ‘mama’ which means mother. They're not my biological mother, but I can call them ‘mama’ or ‘mother’ instead of calling them aunt.” Participant 2 continued and explained that “Even though we're maybe distant cousins – whether it's first, second, third, or fourth cousins – most of the time we would refer to each other as brothers and sisters.”

When it comes to genetics, biological family relationships are important to know in order to establish possible inheritance patterns. When asked how to discern biological family relationships in clinic, Participant 2 stated, “That's why you really have to distinguish to [the patient] what you're looking for and say, ‘You know, I’m trying to find out who your immediate family members are.’” Participant 1 agreed and said, “You have to explain why first. Why are you asking all of these questions? And then we will be more open to tell you.”

### 3.3 Themes addressing aim 2

The second aim of the study was to identify the obstacles in obtaining accurate family health histories, and all participants agreed that language is a major barrier present between patients and providers. The majority of participants indicated that the language barrier alone was sufficient to prevent Marshallese patients from visiting the doctor. In fact, before going to visit a certain clinic or physician, patients want to know what kind of interpretive services are provided at the facility. Participant 3 explained, “The first thing [someone looking for a doctor will] ask me is ‘Do they have interpreters? Do they have Marshallese [*sic*]?” This statement is interesting as it shows there may not be a trained Marshallese clinical interpreter available at the clinic, but in the event that an interpreter is present, patients are likely to feel more comfortable visiting that facility. Study participants indicated that patients often skip appointments when interpreters, or any Marshallese staff are not available to assist them through the clinic visit.

There was also consensus regarding how Marshallese patients interact with and view their healthcare provider. Study participants indicated that the Marshallese tend to interpret and assign more weight to nonverbal cues, such as body language and vocal tone. When asked what behaviors healthcare professionals should avoid, half of the participants said that negative body language will prevent patients from seeking care from that provider in the future. Participant 1 concluded “I think [the providers] get impatient, they get irritated,” which the patients pick up on, so “[The patient] will just let [the provider] talk, and they'll leave, and you'll be lucky if they come back.” Another participant told a story in which she was assisting in a session, and the physician was examining a patient and made a face of disgust when observing the patient's foot. The participant mentioned “The wording were [*sic*] really nice. Good words, kind words. It's just the body language was not there. And then the patient was being withdrawn. Cover [*sic*] it up” (Participant 6). Unfortunately, these kinds of unintended communications can affect whether or not patients return to see the provider in the future.

Word of mouth is the primary way in which many Marshallese learn about clinics and providers available to community members. Part of the information shared with other community members is the overall experience they receive at facilities and with providers. This communication influences the medical decisions of others in the community. Participant 3 mentioned, that “The people who are actually Marshallese community [*sic*] will ask you, ‘Have you been [to a given provider] before?’ And they'll say, ‘Yeah I’ve been there before.’ And the first question is like ‘are they kind?’” Before seeing a provider, the participants stated that Marshallese patients will try to assess the physician to determine whether or not they will feel comfortable going to that clinic and seeing that provider. Participant 4 explained that Marshallese individuals also discuss negative experiences with one another, and if a Marshallese patient is “not comfortable from the start…and then they are seeing how they are being treated, obviously they're not going to want to go back there or they might tell other people, ‘Oh, you don't want to go to that clinic.’” Through the interviews with informants in this study, it became clear that one good or bad experience for a single Marshallese patient can have a large impact on the entire community. Participant 3 stated, “Information travels through the community through word of mouth.”

One practice that is commonplace in the American healthcare system is asking the patient why they are coming to clinic that day. American healthcare providers do this to assess the patient's understanding of why they are being seen and give the patient the opportunity to share current concerns with the provider. However, in Marshallese culture, this question is interpreted as a lack of preparation by the provider. The patient may also downplay their sickness to the provider, instead of being truthful about how they are feeling. Participant 6 explained that Marshallese patients “aren't going to say ‘okay, I’m sick.’ They tend to answer you what you want to hear instead of what they really need.” The participant also contrasted the start of a Marshallese session, explaining the provider should start by saying “‘Okay, so you're diabetic. You came here to see me because…’ They start the conversation with letting them know that [they] already know who you are and [they] already know what's wrong with you.”

Another major barrier in the Marshallese accessing American healthcare is the difference between the healthcare systems in the U.S. and the R.M.I. When describing the Marshallese healthcare systems, it became apparent that the American healthcare system is more complicated and confusing. Participant 5 mentioned that “…when [Marshallese patients] come to the hospital, they don't understand the meaning of appointments,” which can be an issue in the American healthcare system. It is not uncommon for a Marshallese patient to show up much later for their appointment and not realize that they were only scheduled for a given time slot. According to Participant 2, in the R.M.I. “there's no schedule. You go there, and you wait your time, your number to be called [*sic*], and you see the provider at that point.”

Importantly, healthcare financing is one of the key differences between the two systems. Participant 2 explained that in the R.M.I., a patient “can go and see a doctor, [he/she] can get the medications, [he/she] can get [his/her] blood work, and [his/her] blood work, and [his/her] x‐rays, and five or six medications, and it'll all cost [him/her] $5 for that visit.” When these same patients seek care in the U.S., the cost structure, requirements for appointments, and specialists (versus primary care providers) are unexpected and confusing for many. Participant 2 continued that it can be a challenge “for us to come to a system like [the U.S.] …and most of the Marshallese here in Northwest Arkansas are insured… [and yet they] have no money to pay for the services.”

In fact, payment difficulty can be one of the several reasons that a patient does not show up to future appointments. Participants noted that the Marshallese have come to be known as individuals who frequently do not show up to their medical appointments. Participants also noted that this is not because they do not care about their health. Their inability to pay or even lack of realization that a payment is in fact due, can lead to troubles with their insurance or the hospital itself. Participant 5 described how patients may “go to Northwest hospital and get a bill from there, and maybe they lost their job and they don't know that they have to keep up with paying the bills, and they don't know that Northwest [hospital] is going to take them to the court and file a case against them.” This participant also explained how patients may show up for an appointment, realize they owe money from previous appointments and not be able pay, and therefore, cannot be seen.

### 3.4 Themes addressing aim 3

When asked if there are any Marshallese phrases that are important for a healthcare provider to know, all participants agreed that a simple Marshallese greeting would be the best way to start building a relationship between patient and provider. This phrase is, “*iakwe*,” [pronounced YOK‐way] and is typically used by Marshallese when greeting one another. Although one word clearly would not eliminate the language barrier, the participants said learning the phrase demonstrates respect. Participant 2 explained, “You respect us enough that you're trying to learn our language, and hopefully you'll learn the culture as well.” Participant 4 agreed, saying that learning the phrase “*iakwe*” “would give [the patient] maybe a warm welcome feeling of, ‘Oh, I’m being welcomed here, so I can come here. I can tell my other family, “Oh, they're so friendly. They can help you out. They really take good care of you.”’” Learning one phrase in Marshallese shows the patient that they provider is at least acknowledging their language and culture, and from the second quote, it is clear this can have a lasting effect on the patient and the community.

Several participants also noted that American healthcare providers can often be too direct at the start of the session, which can be off‐putting to Marshallese patients, who are typically more conservative. The participants suggested that a bit more small talk at the beginning of the session could be beneficial for the patients to become more comfortable discussing private matters. Participant 5 explained that where she works, students are required to learn a few more Marshallese phrases so that they can make idle conversation at the start of the session. Some of these phrases include: “*Ej et am mour?*” (How are you?); “*Eta in*” (May name is); “*Etam?*” (What is your name?); “*Itok*” (Come, follow me); and “*Jerammon*” (Have a blessed day). As previously mentioned, Participant 1 indicated that providers may become impatient with Marshallese patients and, instead of talking with the patient to uncover why they may not be taking medicine as prescribed or showing up to appointments, the provider will take a more direct, authoritarian tone with the patient. The participant mentioned, “Being too direct with the Marshallese can be not – it's not polite…and so those are times when I see the Marshallese patients, you know they start shutting down.”

The participants agreed that best practice is to utilize an interpreter. One participant went further to suggest a community health worker (CHW) would be most beneficial. A CHW is a Marshallese person who is knowledgeable about the Marshallese community, has been specifically trained for the position, and can assist patients during the session. CHWs are able to interpret the spoken word of the patient and provider but can also interpret the body language and physical cues that each are providing. CHWs can also follow up with the patient in their home or community. Participant 5 explained the difference saying:

A community health worker and the interpreter, they are two different things. Because the interpreting [*sic*], you are supposed to just interpret exactly as the doctor said. But as a community health worker, you have the right to step in and say – when you see your patient is not understanding or your patient, they don’t agree with what you see, but they are not telling the doctor.

This participant stressed the importance of having a CHW present to address nonverbal cues that both the patient and the provider may be expressing. Participant 2 agreed saying CHWs are crucial as they are someone “who speaks [the patient's] language, understands the culture, and can advocate for the patients. And not just interpret, but actually do follow‐ups and kind of make sure they *do* [emphasis added] go to where they need to go.”

Many participants stated that CHWs help facilitate the entire clinic or hospital experience. As previously mentioned, the American healthcare system differs greatly from that of the Marshallese system, so seemingly simple tasks can be confusing or challenging to patients. Participant 4 noted that when entering the facility, patients may not know where or how to sign in, so the hospital should “hire a CHW or something that could help [the patients] out up front, or maybe a receptionist [who speaks Marshallese].” Although it is legally required for every non‐English speaking patient to have access an interpreter during their sessions, whether it be over the phone or in person, it is not required for them to have assistance before and after their allotted time with the provider. Participant 1 explained that when first arriving at the clinic “the patients come in, they still need that person to interpret, and filling out the paper[work].” The participant continued, “I’ve seen where your patient comes in and they just [say] ‘here's the paper work,’ but they still – they wait [to complete it] until they get into the room with the doctor and then they provide the interpreter.” This example highlights the fact that assistance is needed throughout the entire clinical process in order to ensure patients have full comprehension.

Some of the participants also mentioned that a Marshallese patient is more likely to go see a provider if they have met that person prior to the appointment at the facility. Again, this indicates that the provider cares enough to take time out of their day to get to know their patients outside of the clinic setting. Participant 1 suggested that if the provider goes “…out into the community to show that they are the face of [their] organization… then the community can start trusting them.” This participant continued that if a provider goes to Marshallese events, word will spread. “It's not your flyers or [*sic*] it's not what's on TV. But once a Marshallese family starts trusting the organization, then we'll start bringing people in and helping other people to come to your organization.” Trust was a major theme that was consistently brought up in every interview. Attempting to know the patients on a more personal level appeared to be important to building this trust.

Privacy was also frequently mentioned in the interviews. When seeing a physician in the American healthcare system, the provider will ask a multitude of questions about the patient's current and past health concerns. Participant 1 gave an example when having a teeth cleaning and spoke to the dentist about how patients were filling out a questionnaire about their health, and the dentist noted that despite “the high numbers of diabetes in the Marshall Islands…most of them will say, ‘Nope. No.’ to diabetes [in their personal or family history].” The participant then explained “They're there for dental care, [and] that has nothing to do with diabetes,” so they were not providing that information to the dental provider. Since the patients did not understand the correlation between diabetes and dental care, they did not answer the question honestly, thinking that it was not related to their actual health concern.

The importance of privacy is further exemplified when it comes to having a provider of the same gender caring for the patients. This matter was brought up by several participants, include Participant 2 who explained, “Anything in the genital area and in the private parts, it's very sacred for us. And we don't discuss that. We don't even talk about that in front of our opposite sex relatives in the room.” This participant also gave an example of how this can be an issue in the healthcare setting. Although it is important to have the same gender provider and patient, the interpreter must also be of the same gender. The participant mentioned, “If you're using an interpreter, or a translator, interpreting their room, it's gotta [*sic*] be of the same sex, that you're asking those kind of sensitive kind [*sic*] of questions.” This can prove to be a great barrier in healthcare access, as some patients may not feel wholly comfortable being cared for or talking about personal matters with a person of the opposite gender.

Participant 2 suggested another way to address personal matters with participants during the physical examination. The participant explained, “Any time that you ask questions, you should always say ‘I’m sorry if this is insensitive’…especially if you don't know if it's appropriate or not, apologize before you ask the question. Or apologize before you even touch the patient.” Apologizing beforehand demonstrates respect and makes the questions and physical examination seem less intrusive. The participant continued saying that if you do not ask these questions beforehand, the patient will still let you examine them, but may not be willing to return. The reaction from the patient differs slightly when asked personal questions, however. If the provider does not apologize before asking these questions, the patient may stop answering truthfully, and instead just tell the provider what they think the provider wants to hear. In order to elicit accurate information from patients, providers need to be polite and apologize before asking for intrusive information.

## 4 DISCUSSION

The current research study identified themes unique to the Marshallese population that are valuable to genetics providers working with this population. In addition to the population‐specific findings, the identification of fluid family dynamics and the barriers that minorities face when seeking healthcare in the U.S., were two conclusions consistent with findings identified in studies of other minority populations (Reinschmidt et al., 2015; Weaver & White, 1997; Williams & Hampton, 2005; Wong et al., 2017). However, this study identified other themes that were unique to the Marshallese population and important for genetics providers.

### 4.1 Themes addressing aim 1

The first theme identified that addressed aim one was the importance of large extended families in the Marshallese community. Based on previous conversations with personal contacts, as well as similar findings in studies about the family relationships of other populations, these results were expected. During one conversation, it was explained that the Marshallese sense of family was similar to that of Native Americans (N. Aitaoto, personal communication, March 16, 2017). In fact, Weaver and White (1997) noted that Native American families feel a strong sense of relationship with the seven generations before them and seven generations to come. This finding is similar to those identified in this study, in which a participant noted the Marshallese extended family goes back four or five generations.

Similar to findings of White and Weaver (1997) is the sense of identity belonging to the land. In the case of the Marshallese, they identify with their home island or atoll, while Native Americans have a close sense of pride with their home tribe and the land where they were raised. The current study parallels that of White and Weaver (1997) once again because the findings both stated that families can include nonbiologically related individuals and can include people who are close to you and may have grown up around you as well. The findings on child‐rearing in the populations of each of these studies also showed similarities, in that raising a child is not just the responsibility of the biological mother and father. The current study, however, found that the view on adoption was unique, in that the Marshallese population is more likely to allow another member of the family raise the child as their own. This information may also be difficult to elicit while taking a FHH from a Marshallese patient without the provider knowing the nature of Marshallese family dynamics.

Another unique finding in this study was the difference in terms used to reference family members. The current literature did not provide any examples of other communities using terms such as “mother,” “father,” “sibling,” and “cousin” with such fluid meaning as the Marshallese population. This study found that even biological cousins may be referred to as brother and sister, while the women who help raise a child may be referred to as mother, despite the actual biological relationship. The close‐knit nature of Marshallese families can create difficulty when trying to assess family relationships, which is important when eliciting and interpreting a FHH.

The genetic counselor or other provider who is taking the FHH and asking questions about family history should be cognizant of the way the Marshallese may speak of family members. In order to do this, the current study found that the genetic counselor should be very specific when asking about the relationships between the patient and other family members. It also found that it is important for the genetic counselor to state why all of the FHH questions are important, and how they directly relate back to the patient's own health. This way, the patient will be more willing to provide accurate information with the understanding of how this information is relevant.

### 4.2 Themes addressing aim 2

When it came to identifying the barriers that impact obtaining a FHH, many of these findings were also expected. Initially, participants explained that language barriers can be an issue for many Marshallese patients, which was unsurprising, after the 2005 study by Williams and Hampton reported the same information. This is important for genetic counselors, since, as mentioned before, this study identified certain barriers that are directly applicable to family relationships.

One somewhat surprising fact uncovered here is the power of information that is spread by word of mouth through the community. Although it is common for members of all communities to share information about past experiences with providers, this study found that sharing information within the Marshallese community is much more powerful. Participants explained that it only takes one positive or negative experience by one Marshallese individual to build a reputation throughout the entire community. This is important for genetic counselors to note and could be the reason why Marshallese patients visit a specific provider's clinics more or less often.

Another commonality between the Marshallese community and many other communities is making note of the provider's tone of voice and body language. Although it is common for all humans to acknowledge the body language of others, since there is a spoken language barrier between the Marshallese patients and their providers, a Marshallese patient may read further into body language than a patient who speaks the same language as their provider. This information is helpful for providers to remember when working with Marshallese patients.

The drastic differences between the American and Marshallese healthcare systems can be a barrier to attending specialty appointments such as genetics. Moreover, if a Marshallese patient keeps the genetics appointment, the barriers identified in this study could affect the patient's follow through with recommendations, which may include evaluations by other specialists, as nearly every specialty will require a separate appointment, likely on a different day, and possibly even a new location. Finally, genetics evaluations are often consultative in nature, so it may also appear that the geneticist/genetic counselor does not “do anything.”

### 4.3 Themes addressing aim 3

This study also identified communication strategies that can be used to elicit an accurate FHH. It found that the first impression between patient and provider is crucial for a successful, long‐lasting relationship. Genetic counselors should begin each session by saying “*iakwe*” [pronounced YOK‐way] to start to earn the patient's trust. Creating a positive first impression will also be beneficial to earn a good reputation among the entire community, which was an important aspect mentioned in the themes addressing Aim 2. Part of creating a good first impression and relationship is also not to be too direct with a patient and begin with some small talk. Genetic counselors are trained to contract with their patients, in order to build rapport and negotiate what needs to be accomplished during the appointment, so counselors should take special note when seeing Marshallese patients, as contracting may be crucial to establishing a positive relationship. However, another common practice in genetic counseling is asking the patient about their understanding of the genetic appointment. It is not uncommon for genetics patients, in general, not to fully understand the reason for referral. With Marshallese patients, however, asking the patient why he or she is there will seem as though the genetic counselor is not prepared for the session and truly does not know why the patient has shown up. The study found, that the best approach is for the genetic counselor to review the reason for the referral and explain the purpose of the appointment with the patient before asking the patient open‐ended questions that explore their concerns.

Another very helpful strategy uncovered here is the use of a CHW. Although interpreters can be very useful when a CHW is not available, the skills and training that a CHW has are incredibly helpful in a medical setting. Not only can the CHW help interpret during the session, but also they can assist with nonverbal communication. If the CHW recognizes that a patient does not understand a certain concept, but is nevertheless nodding his/her head in understanding, the CHW can interject and inform the provider and/or encourage the patient to ask their questions or share their concerns. Culturally, the Marshallese people defer to authority and tend not to assert or advocate for themselves. If the provider has unknowingly offended the patient, or is not clearly explaining the information, it is highly unlikely that the patient will interrupt, inform the provider, or seek clarification. Rather, a typical response would be to nod in agreement and appear understanding and compliant. Marshallese CHWs know this, of course, and bridge the total communication for patients and providers.

The benefits of CHWs have been seen in other minority populations in the U.S., including the Hispanic population. In a study by (Reinschmidt et al., 2015), thirty‐eight interviews were conducted with CHWs and organization leaders of a Hispanic advocacy group in Arizona. They concluded that patients were not receiving complete care without the assistance of CHWs who served as advocates and educators for patients (Reinschmidt et al., 2015). Healthcare providers should be aware of the benefit of using a CHW when serving their Marshallese patients, since it has proven to be essential to the proper care of minority populations in the U.S. Unfortunately, there is not an abundance of CHWs to serve the growing population in the U.S., so facilities may want to consider connecting with distant CHWs over telecommunication devices, as they have specific training and knowledge to best assist Marshallese patients.

This study identified that privacy is very important to the Marshallese community, including withholding personal and family health information from healthcare providers who have not yet earned the patient's trust. Due to past negative experiences with American healthcare providers, Marshallese patients are not always willing to give providers their health information, which can be difficult when genetic counseling relies so heavily on patient reported history. In order to best obtain this information, the genetic counselor should build a strong relationship from the start with their patients to start to earn their trust.

This study also found that it is important for the provider and patient to be the same gender. The issue of having a patient and provider who are the same gender can also be complicated when it comes to genetic counseling. The vast majority of genetic counselors are female, so ensuring the gender of the patient and provider are the same can be a challenge. In this study, we did not identify what to do if there is not a same‐gendered provider available and could be further explored in another study.

## 5 CONCLUSIONS

The population of Pacific Islanders, and notably Marshallese, is rapidly growing in the U.S., and specifically Northwest Arkansas (McElfish et al., 2017). The initial migration occurred after the nuclear weapons testing program exposed the islands to radiation (Barker, 2004), however, changing climate threatens to completely destroy the low‐lying Pacific nations (Patel, 2006). As a nation with an average elevation of seven feet above sea level (Barker, 2004), climate change poses a great risk to flood or completely destroy the R.M.I. With a disappearing land mass and the COFA in place, the population will only continue to migrate to the U.S. (Davenport, 2015).

Although limited literature exists about how to address other ethnic populations in healthcare, there is little information published about Pacific Islanders, and more specifically the Marshallese. With the extensive health issues that the Marshallese are living with, healthcare providers will increasingly encounter this population in American hospitals and clinics. This study's findings are especially relevant for genetic counselors as we have identified new strategies to obtain a FHH from the Marshallese population, which is essential to the success of genetic counseling and a genetics evaluation for this population.

This study found a few strategies for genetics providers to use including: (a) attend Marshallese events to earn the trust of the community; (b) start the session with small talk; (c) explain to their patients why they are asking the family health questions; (d) be as specific as possible when asking about the patient's relationship to different family members to discover the true genetic relationship; and (e) ensure a CHW is present at the session for interpretation of verbal and nonverbal information. Appendix C in the Supplementary File contains specific tips and strategies for American providers who may encounter Marshallese patients.

### 5.1 Study limitations

The themes identified in this study are limited to the study population, which was composed of a small sample size. Although Marshallese individuals were contacted with a variety of backgrounds, the majority of the study respondents (five of the six) were medical professionals, which may be attributed to ascertainment bias. It must be acknowledged that the responses to the questions may be the result of professional bias based on experiences. The study also only consisted of individuals currently living in Northwest Arkansas, so there may be some bias relating to their location.

### 5.2 Research recommendations

As mentioned previously, there are several findings that should be explored further, including the Marshallese understanding of genetics as a whole and identifying culturally appropriate concepts that equate to key concepts in genetics. Exploration into the identification of genetic disorders or variants more common among the Marshallese population due to geographical isolation and possible lack of intercultural reproduction are limited. Noting the study limitations, additional studies are warranted with participants from a variety of professional backgrounds and from the general community. This will hopefully eliminate the potential professional bias of the findings in this study. Although the population of Marshallese in the U.S. is greatest in Northwest Arkansas (McElfish et al., 2017), a similar study could include Marshallese participants from all over the country to determine if the results compare. Including more states could ensure that the results of this study represent all Marshallese populations living in the U.S. Finally, it would be interesting to learn how Marshallese patients respond to telehealth modalities for accessing genetics services. Telehealth could allow healthcare providers and CHWs to assist more patients across the country. Finally, another study could be conducted on the health literacy in the Marshallese population, as well as identifying strategies to educate this population on genetics concepts.

## AUTHOR CONTRIBUTIONS

Karli Blocker, Lori Williamson Dean, and Noelle R. Danylchuk conceived and developed the research. Karli Blocker acquired the data, while she and Lori Williamson Dean then interpreted and analyzed all of the data. Karli Blocker, Lori Williamson Dean, and Henry Gene Hallford drafted the manuscript, with assistance from Dr. Pearl McElfish and Noelle R. Danylchuk. All authors approved the final draft of the manuscript and agreed upon its contents.

## CONFLICT OF INTEREST

Karli Blocker, Henry Gene Hallford, Pearl McElfish, Noelle R. Danylchuk, and Lori Williamson Dean declare that they have no conflicts of interest.
