# Supplementary material for: Eliciting culturally and medically informative family health histories from Marshallese patients living in the United States
Source: J Genet Couns. 2020 Apr 28;29(3):440–50. doi: 10.1002/jgc4.1249 (PMC7318156; doi:10.1002/jgc4.1249)
Supplement: Supplementary file 1 [file JGC4-29-440-s001.pdf]

APPENDIX A

INTERVIEW GUIDE FOR KEY INFORMANT INTERVIEWS

My name is Karli Blocker and I am studying at UAMS to get my Master's degree in Genetic Counseling. I would like to inform you that I will be reading a script, so some of the language will come across as unnatural. We are conducting a study to better understand how health care professionals can interact with Marshallese patients to improve their experience at American hospitals and clinics. We also hope to come to a better understanding of how Marshallese individuals refer to biological family members, in order to get a more accurate medical history at American hospitals and clinics. You may have answered some of these questions before, but we are using this information to discover how genetic counselors can better interact with their patients.

Thank you for your willingness to be interviewed and for participating in this research project. Do you have any questions about the consent from that was sent to you?

Now we have reviewed the informed consent document and you are aware of any potential risks or benefits. Can you please confirm that you have reviewed the form and you give your consent to participate?

You have the ability to skip any of the questions that you don't want to answer. Remember we can stop at any time if you feel uncomfortable. Do you have any questions at this time?

I would just like to remind you that this interview is being recorded.

Personal History

So, I would like to start with a question about you. Can you please tell me a little about yourself?

What do you do for work? What do you do in the community?

Family Health History

What is family to you? How do you define family? Who are the members of your family? When you use the word family, who are you talking about?

Do members of the Marshallese community have ways or phrases to differentiate blood relatives or "kin" from adopted relatives? If yes, please explain.

If the doctor wants to know information about blood related relatives, how should the doctor or nurse ask those questions?

When doctors or nurses ask patients (like you) about biological or blood relative's health information, how should they ask that question?

- Do you have any suggestions for how to explain why the doctor or nurse is asking these questions?

If the child is not biologically related to the parents (not related by blood), how should the doctor or nurse ask those questions? (If the child is adopted, how should a doctor or nurse ask about the child's biological family?)

Personal Experience

Please tell me about your personal experiences with American hospitals and clinics. For example, did you leave feeling satisfied or fulfilled? Did you have any negative thoughts when you left? Were the doctors and nurses being helpful?

- Describe your positive experiences with the American hospitals and clinics, if any.
- Describe your negative experiences with the American hospitals and clinics, if any.

Creating a Relationship

What can a doctor or nurse do to be respectful, earn your trust, and create a long-term relationship?

- Are there any specific greetings, phrases, introductions, or things that doctors or nurses need do to make you feel more comfortable?

Are there any behaviors or actions a doctor or nurse should avoid, as it may be culturally insensitive or frowned upon in the Marshallese community?

I would now like to ask you questions about the relationship between the US and Marshall Islands that is related to the bombings, would that be okay?

Do you think that the bombings in the Marshall Islands between 1946-1958 had negative health effects? If yes, how?

- Do you think this affects the Marshallese from going to an American hospital?
- If yes, what can the doctor do to reduce this hesitancy?

Marshallese seeking health care

In general, do you think Marshallese are hesitant to seek health assistance from the U.S.?

- Can you tell me more about that?
- What can the doctor or nurse do to make the experience better for the Marshallese patients?

(Do you think there are barriers between Marshallese patients and American health care providers?) – may get the answer before hand

- Can you tell me more about that?
- What can health care providers do to remove these barriers?

What can health care providers do to encourage Marshallese individuals to go to the doctor when they are ill?

- Would more services or interpreters make them feel more comfortable going to the American service provider? If so, what are these services?

APPENDIX B

MARSHALLESE TRANSLATIONS

Marshallese word	Pronunciation	English translation
ajej in mona	AH-jeej EN MOH-nah	given as food
baba	BAH-bah	father
bar iakwe	BAR YOK-way	goodbye
Ej et am mour?	EHJ EET OHM MOOR	How are you?
ek in karok	EK EN car-ROK	fish contaminated by radiation
Eta in...	EH-tah IN	My name is...
Etam?	EH-tahm	What is your name?
iakwe	YOK-way	hello; greeting
Inaaj ajej in mona ki woj ajiri e neju	en-AWHJ AH-jeej EN MOH-nah KEE WAHJ AHJ-ee-ree NEH-juh	I will pass my child as food over to you
itok	ee-TAHK	come
jeimjatin	gem-JAH-tin	sibling; brother; sister
jerammon	jeh-RAHM-on	go in peace
jerammon ilo rainin	jeh-RAHM-on EE-lo RAH-nin	have a blessed day
jokon	jo-KAHN	cane
kajiriri	kah-JEH-ree-ree	person who is adopted
leo belele	LO BAH-lay-lay	husband
lio belele	LEE-oh BAH-lay-lay	wife
maanim	MAH-nim	cousins whose parents are the same gender
mama	MAH-mah	mother
meanwod	meh-OHN-wood	humble; patient
neju	NEH-juh	child of; son of; daughter of
rilikim	REE-lee-kim	cousins whose parents are opposite genders
weto	WAH-too	piece of land

APPENDIX C

TIP SHEET FOR HEALTHCARE PROVIDERS

Quick Tips for American Providers Who Serve Marshallese Patients

What should healthcare providers do or understand?	Why is this helpful or important?
☐ Say "*iakwe*" [pronounced YOK-way] at the start of the appointment.	☐ Shows that the provider cares enough to learn something about the patient's language and culture.
☐ Make small talk with the patient before discussing medical matters.	☐ This helps build the patient-provider relationship and the patient will begin to trust the provider.
☐ Marshallese families are very large and include non-biological family members.	☐ Providers should be aware for this in order to ask the appropriate questions when taking a family health history.
☐ Explain why every family and personal health history question is being asked.	☐ Patients will be more willing to give accurate information if they understand how it affects their health.
☐ Have a community health worker present at the clinic, and ensure they are accessible to the patient from the start to the end of the appointment.	☐ The Marshallese and American healthcare systems are different, and common practices in American facilities may be foreign or confusing to Marshallese patients.
☐ The Marshallese have strong faith and belief in God which may lead them to seek a combination of healthcare services.	☐ Providers should respect a patient's beliefs and recognize they may choose to seek spiritual guidance, Western medicine, and/or traditional medicine.
☐ Healthcare providers are encouraged to go to Marshallese events to meet members of the community outside of the clinic or hospital.	☐ Marshallese patients will begin to trust providers if the provider makes an effort outside of the clinic.
☐ Marshallese patients will read the body language and tone of voice of providers.	☐ The providers can make an additional effort to be aware of their body language and tone.
☐ Don't ask the patient why they are at the appointment.	☐ Patients will think that the provider has not reviewed their chart and may not be prepared for the appointment.
☐ The Marshallese are very private people and do not speak of private matters in front of people of the opposite gender and family members.	☐ The provider, patient, and CHW should all be the same gender. The provider should also ask the patient if it is okay to discuss private matters in front of everyone in the room.
☐ The provider should apologize to the patient before performing the physical exam.	☐ Since the Marshallese are such private people, touching or examining without apologizing can be offensive.

Property of Heartland Regional Genetics Network and the University of Arkansas for Medical Sciences
Created by Karli Blocker, Gene Hallford, Nia Aitaoto, Noelle Danylchuk, and Lori Williamson Dean

Tip Sheet for American Providers Who Serve Marshallese Patients

What can a healthcare provider say to a Marshallese patient to make the patient feel more comfortable?

- Say "*iakwe*" [pronounced YOK-way] at the start of the appointment.
    - *"Iakwe"* is the Marshallese word for hello. Saying this simple phrase shows the patient that the provider cares enough about the patient to learn something about the patient's language and culture.
- Make small talk with the patient before discussing medical matters.
    - This helps the patient start to trust the provider. Talking about non-medical matters shows that the patient cares about the patient on a more personal level.

What services can a healthcare facility, hospital, or clinic provide to encourage the Marshallese population to seek healthcare when ill?

- A Marshallese community health worker (CHW) should be present at the facility and available during the patient's entire visit.
    - A CHW can interpret both spoken language and non-verbal cues between both the patient and provider, which can help break down the language barrier. The CHW should also be present for the whole visit, including check-in or registration, filling out paperwork, the appointment, making follow-up appointments, filling prescriptions, and the payment process, as these procedures may be unfamiliar to Marshallese patients.

What should healthcare providers understand about Marshallese family relationships when taking a family health history?

- Marshallese families are very large and include non-biological family members.
    - When asked about family members, Marshallese patients may provide information about individuals who are not blood related to them.
- The Marshallese may use the word "brother" or "sister" as a word to describe their siblings, cousins, or close friends.
    - Because of this, the exact biological relationship should be explicitly explained when taking a family health history. Similarly, when asking about the patient's mother, the patient may provide information about the person who played a maternal role but instead of the biological mother.

What should healthcare providers understand about Marshallese culture in order to better serve their Marshallese patients?

- The Marshallese value a healthcare provider who attends events in the Marshallese community and attempts to meet them before they are in a healthcare setting.
    - This will help the community build trust in the providers and be more willing to seek healthcare at American facilities.
- Marshallese people are very private and may not provide accurate or thorough information when questioned about their personal and family health histories.
    - The provider should explain why they are asking these questions and how they pertain to the patient's health in order to obtain the most accurate information.

Property of Heartland Regional Genetics Network and the University of Arkansas for Medical Sciences
Created by Karli Blocker, Gene Hallford, Nia Aitaoto, Noelle Danylchuk, and Lori Williamson Dean
